# Emotion Dysregulation, Impulsivity, and Implicit Cognition in Adolescent with Self-Injurious Thoughts and Behaviors: A Six-Month Prospective Study

**DOI:** 10.3390/jcm14248705

**Published:** 2025-12-09

**Authors:** Inmaculada Peñuelas-Calvo, María Taracena-Cuerda, Manon Moreno, Sandra Cabrera-Redondo, Vera Álvarez-González, Rodrigo Puente-García, Blanca Quintana-Saiz, Ana Jiménez-Bidón, Alejandro Porras-Segovia

**Affiliations:** 1 Department of Child and Adolescent Psychiatry, Hospital Universitario 12 de Octubre, 28041 Madrid, Spain; inmaculada.penuelas@salud.madrid.org (I.P.-C.); sandra.cabrera@salud.madrid.org (S.C.-R.); vera.alvarez@salud.madrid.org (V.Á.-G.); blanca.quintana@salud.madrid.org (B.Q.-S.);; 2 Health Research Institute Hospital 12 de Octubre (i+12 Institute), 28041 Madrid, Spain; 3 Department of Psychiatry, Universidad Complutense de Madrid, 28040 Madrid, Spain; 4 CIBERSAM-ISCIII (Biomedical Research Networking Centre for Mental Health/Carlos III Health Institute), 28029 Madrid, Spain; 5 Department of Psychology, Universidad of Villanueva, 28039 Madrid, Spain; 6 Mental Health Research Group, Health Research Institute Jiménez Díaz Foundation, 28040 Madrid, Spain; 7 Department of Psychiatry, Hospital Universitario Rey Juan Carlos, 28933 Móstoles, Spain

**Keywords:** psychological pain, suicidal behavior, adolescents, impulsivity, trauma, implicit association test

## Abstract

**Background/Objectives:** Suicide is a leading cause of death among adolescents. Emotion dysregulation, impulsivity, and childhood trauma are key factors underlying Self-Injurious Thoughts and Behaviors (SITB), yet reliable short-term predictors are limited, especially in at-risk clinical populations. This study prospectively examined the association between SITB and clinical (psychological pain, impulsivity, childhood trauma) and cognitive measures (Implicit Association Tests, IATs). **Methods:** A prospective, observational study was conducted in adolescents (12–17) admitted to a university hospital psychiatry unit following recent SITB. Participants completed the Death/Suicide IAT (D/S-IAT), Self-Injury IAT (SI-IAT), and standardized instruments including the Columbia Suicide Severity Rating Scale (C-SSRS), Difficulties in Emotion Regulation Scale (DERS), Barratt Impulsiveness Scale (BIS-11), and Childhood Trauma Questionnaire (CTQ-SF). SITB recurrence was assessed at six months. **Results:** Within six months, 28.9% of 38 participants reported suicidal thoughts, 15.8% engaged in self-injury, and 2.6% attempted suicide. The SI-IAT showed a small but significant correlation with C-SSRS, whereas D/S-IAT showed none. Neither IAT predicted SITB recurrence. Higher levels of emotion dysregulation and impulsivity were significantly associated with SITB. Specific DERS dimensions—emotional rejection, interference, and confusion—predicted future SITB, highlighting emotional dysregulation as a vulnerability factor. **Conclusions:** In high-risk adolescents, psychological pain and impulsivity predicted SITB more reliably than IATs. Unlike adult populations, explicit measures outperform implicit ones in suicide risk prediction. These findings emphasize emotion dysregulation as a key clinical construct that may intensify psychological pain and contribute to suicidal vulnerability.

## 1. Introduction

Suicide is one of the leading causes of unnatural death worldwide, accounting for more than 700,000 deaths each year [1]. It is the third leading cause of death among 15- to 29-year-olds [1]. The lifetime prevalence of suicidal thoughts in children and adolescents is estimated at 18%, and of suicide attempts at 6% [2]. These attempts are approximately 20 times more frequent than deaths by suicide [3], and those occurring at an early age are associated with a higher risk in adulthood [4].

Self-Injurious Thoughts and Behaviors (SITB) have a multifactorial origin, influenced by biological, psychological, social, and environmental variables [5]. Childhood and adolescence are critical periods of development during which exposure to adverse factors can significantly increase the risk of suicide [3,5]. One of the most studied of these is childhood trauma, which affects up to 30% of the general population [6,7]. Empirical evidence has demonstrated a consistent relationship between these experiences and the subsequent development of SITB [8].

Emotion dysregulation refers to difficulties in identifying, understanding, and managing emotional experiences. It includes problems such as heightened emotional reactivity, impulsive responses to distress, and limited access to adaptive regulation strategies. In adolescents, emotion dysregulation has been consistently associated with the onset and recurrence of SITB [9,10]. This construct is closely related to, but distinct from, psychological pain or *psychache*, as difficulties in emotion regulation can intensify subjective distress and feelings of inner suffering. In this sense, emotion dysregulation may contribute to the experience of psychological pain, which has been proposed as a proximal mediator between emotional distress and suicidal behavior [11,12,13].

Moreover, recent research has suggested that implicit cognitive processes—such as automatic self-associations captured by the Implicit Association Tests (IATs)—may interact with emotion regulation mechanisms [14,15,16]. Impaired emotion regulation could amplify the salience of negative implicit associations related to the self, death, or self-injury, thereby increasing psychological pain and vulnerability to SITB. Conversely, interventions that strengthen emotion regulation abilities may help reduce psychological pain and mitigate suicide risk. Understanding how explicit (emotion regulation, impulsivity) and implicit (IAT-based) processes jointly influence suicidal behavior may provide a more comprehensive framework for prevention and intervention in high-risk adolescents.

Among the various risk factors associated with SITB, childhood trauma stands out as one of the most consistently identified [6]. Childhood trauma encompasses any form of physical and/or emotional maltreatment that results in actual or potential harm to a child’s health or development within a relationship of responsibility, trust, or power. Physical, emotional, and sexual abuse, physical neglect, and exposure to domestic violence during childhood have all been recognized as significant risk factors for later SITB [8]. Furthermore, impulsivity is a significant risk factor for suicide and a recent meta-analysis showed that impulsivity mediates the relationship between childhood trauma and SITB [17,18]. Indeed, maltreatment can affect neurobiological, cognitive, and emotional development, thereby promoting the emergence of impulsivity and increasing the risk of suicide [6].

Although previous research has examined the role of emotion dysregulation, impulsivity and childhood trauma in SITB [6,7,8,9,11,17,18], few studies have simultaneously integrated explicit (self-report) and implicit (IAT-based) measures in adolescent populations [14,15]. Furthermore, while the predictive validity of implicit association tests has been demonstrated in international adult samples, evidence in Spanish adolescents, particularly those with a recent history of SITB, is still limited. This study therefore makes a novel contribution by assessing the combined predictive capacity of explicit and implicit measures in a Spanish adolescent sample. This allows for a more comprehensive understanding of the mechanisms underlying SITB, offering insights that are relevant for national and international prevention strategies [6,7,8,9,10,14,15,17,18,19,20].

However, predicting suicide remains challenging in clinical practice. Unstructured interviews, which are the main method of assessment, have limitations, particularly when patients deliberately hide their SITB. One study found that 78% of people who died by suicide had denied such intentions in a previous assessment [19]. This assessment is usually carried out in emergency settings, where there is added time pressure and workload [20].

In light of these limitations, tools based on implicit cognition have been developed to detect automatic, non-conscious associations that are less susceptible to voluntary manipulation [14]. Such tools allow for the detection of implicit associations between the self and suicide-related concepts. The Death/Suicide Implicit Association Test (D/S IAT), developed by Nock et al. (2010) [15], measures how quickly concept of the ‘self’ are associated with ‘suicide’ or ‘life’. In their study, a strong implicit association between the self and suicide was found to predict suicidal attempts, increasing the risk sixfold in the following six months [15]. This instrument has recently demonstrated predictive validity in a Spanish adult population [14].

Another test, the Self-Injury IAT (SI-IAT), assesses the association with non-suicidal self-injury (NSSI), which is a common behavior among adolescents and closely linked to suicide risk [10]. Despite their potential, none of these instruments has yet been validated for use with the Spanish adolescent population, particularly those with a recent history of SITB, in order to assess their predictive utility in the short term.

This study aims to examine the predictive capacity of explicit (emotion dysregulation, impulsivity, childhood trauma) and implicit (IAT-based) measures in relation to SITB among adolescents aged 12 to 17 with a recent history of these behaviors. Specifically, it investigates whether implicit associations between the self and concepts of death or self-injury predict the recurrence of SITB over a six-month follow-up. Furthermore, it aims to identify whether explicit measures—particularly emotion dysregulation—show greater predictive power than implicit ones.

## 2. Materials and Methods

### 2.1. Ethical Considerations

All procedures adhered to the European General Data Protection Regulation (Regulation EU 2016/679). No identifiable data (e.g., names or personal details) was published in any report or dissemination arising from the study. Each participant was assigned a unique pseudonym to ensure confidentiality. Personal data were stored on secure institutional network drives at Hospital Universitario 12 de Octubre.

Only authorized clinical personnel (psychiatrists, psychologists and nursing staff) had access to this data. Anonymized data could be accessed by research team members or collaborating institutions, but only for scientific purposes. Data will be retained throughout the duration of the study and for up to five years following its completion.

Participants and/or their legal representatives were informed of their right to access, correct or request the deletion of their personal data at any time. The study protocol was reviewed and approved by the ethics committee of Hospital Universitario 12 de Octubre (Madrid, Spain). All participants (or their legal guardians) provided written informed consent prior to taking part in the study.

### 2.2. Study Design

This is a 6-month prospective, longitudinal, observational study examining recurrence of SITB in adolescents. The study was conducted at the Hospital Universitario 12 de Octubre (Madrid, Spain) and approved by the institutional ethics committee. Informed consent was obtained from all parents or legal guardians and the participants.

#### Sample

Adolescents aged 12 to 17 years with a history of SITB within the previous month were included in the study. Participants were recruited from the child and adolescent impatient unit of the University Hospital 12 de Octubre in Madrid, Spain. The inclusion criteria were as follows: (I) age between 12 and 17, (II) having engaged in SITB within the past month, (III) being able to understand the study procedures, and (IV) having fluency in Spanish. Exclusion criteria included: (I) the inability of parents or legal guardians to provide informed consent and, (II) the presence of a psychotic disorder or intellectual disability.

### 2.3. Sample Size Calculation

The sample size was estimated in advance using G*Power 3.1 software. This considered a medium effect size (f^2^ = 0.15), a significance level of α = 0.05 and a statistical power of 80%. This indicated that at least 35 participants were needed to detect significant associations in the regression models.

### 2.4. Measures and Procedure

Once written informed consent had been obtained from both the participants and their parents or legal guardians, sociodemographic data were collected from each participant, including age, sex, and current academic level. Participants then completed two computerized implicit association tests (IATs): D/S-IAT and SI-IAT, followed by standardized clinical scales. All assessments were conducted by trained clinicians or researchers. The evaluation included the following assessments:

#### 2.4.1. Explicit Measures

Validated measures were used to examine suicidality, psychological pain, impulsivity, and exposure to childhood trauma.

The presence, severity, and intensity of SITB were assessed using the Columbia Suicide Severity Rating Scale (C-SSRS) [21]. This semi-structured clinical interview evaluates four primary constructs: the severity and intensity of suicidal thoughts, self-injury and the lethality of suicide attempts. The C-SSRS is widely regarded as the gold standard for assessing suicidality in clinical and research settings.

Emotion dysregulation was measured using the 28-item self-report Difficulties in Emotion Regulation Scale (DERS) [22]. This scale assesses multiple aspects of emotional suffering, including emotional awareness, emotional clarity, non-acceptance of emotional responses, difficulty controlling impulses, limited access to regulation strategies and difficulty engaging in goal-directed behavior during distress. Although the DERS does not directly measure psychological pain, several of its subdimensions, such as non-acceptance of emotions, difficulty controlling impulses and limited access to regulation strategies, capture facets of emotional suffering that may underlie psychological pain.

Impulsivity was measured using the validated 30-item Barratt Impulsiveness Scale, Version 11 (BIS-11) [23], which assesses trait impulsivity. Items are grouped into three subscales: Cognitive Impulsivity (e.g., making rapid decisions), Motor Impulsivity (e.g., acting without thinking), and Non-Planning Impulsivity (e.g., lack of foresight). Responses are rated on a 4-point Likert scale.

Exposure to childhood trauma was assessed using the 28-item Childhood Trauma Questionnaire—Short Form (CTQ-SF) [24], a retrospective self-report measure evaluating experiences of childhood maltreatment. The questionnaire includes five domains: emotional abuse, physical abuse, sexual abuse, emotional neglect, and physical neglect. Items are scored on a 5-point Likert scale ranging from ‘never true’ to ‘very often true’.

#### 2.4.2. Implicit Measures

Two five-minute computerized implicit association tests were administered.

Death/Suicide Implicit Association Test (D/S-IAT): This test assesses the automatic cognitive associations between the self and the concept of death. Participants classify words from semantic fields related to ‘death’ (e.g., dead, dying, suicide) and ‘life’ (e.g., living, surviving) as either ‘self’ or ‘not self’ using binary key responses. Association strength is expressed via the D-score, with a standard cut-off point of 0.00. Positive scores suggest an implicit connection between the self and death, while negative scores suggest a stronger connection with life.

Self-Injury Implicit Association Test (SI-IAT): This test evaluates implicit attitudes towards self-harm. Participants rapidly categorize emotionally salient images (e.g., lacerated skin) and neutral images (e.g., intact skin) under the labels ‘cutting’ and ‘not cutting’. Faster reaction times in congruent pairings suggest a stronger implicit association with self-injurious behavior.

All instruments were administered in a single session. Trials with response latencies below 300 ms or above 10,000 ms were excluded from the analyses. Error trials were adjusted following standard IAT procedures. Internal consistency assessed by split-half reliability was r = 0.78 for the Death/Suicide IAT and r = 0.81 for the Self-Injury IAT. No participants were excluded due to an excessive error rate. At the six-month follow-up, the researchers assessed the recurrence of SITB by conducting a systematic review of the participants’ electronic clinical records. This review documented follow-up appointments and ED visits related to SITB.

### 2.5. Statistical Analysis

Statistical analyses were conducted using the JAMOVI software package (v2.5.5). Statistical significance was set at *p* < 0.05, with two-sided tests. Confidence intervals (95% CI) are reported consistently with both the lower and upper bounds formatted as (lower bound, upper bound). A combination of simple logistic regression and Pearson’s correlation coefficients was employed. To minimize overfitting, logistic regressions were conducted separately for each predictor (bivariate models). The number of events per variable ranged from 6 to 11, warranting cautious interpretation. The gold standard for suicidality assessment was the C-SSRS.

Additionally, binomial logistic regression was performed to examine the relationship between D/S-IAT and SI-IAT scores and the recurrence of SITB at six months, including self-injurious, suicidal thoughts and suicide attempts.

All ORs were unadjusted and expressed per 10-point increase in continuous scales to aid clinical interpretation. This was conducted mathematically by raising the ORs for a 1-point increase to the power of 10.

An a priori power analysis was performed, assuming a medium effect size. Due to the presence of multiple endpoints and predictors, formal corrections for multiplicity were not applied; therefore, the results should be interpreted as exploratory.

## 3. Results

### 3.1. Sample Characteristics

The sample consisted of 38 adolescents (M = 14.92 years, SD = 1.46) predominantly female (94.7%), aged between 12 and 17 years. The most common primary diagnosis was mood disorders (52.6%), while medication overdose was the most frequently reported form of suicidal attempt (23.7%). Sociodemographic variables assessed included, among others, parental divorce (55.2%), family conflict (57.9%), history of abuse (16%), and being a first-generation migrant (18.4%) (see Table 1). Complete follow-up data at six months were available for all 38 adolescents who were initially assessed. During this period, self-injurious behavior recurred in 6 (15.8%) individuals, suicidal ideation was reported by 11 (28.9%) individuals, and one (2.6%) participant made a suicide attempt. These event counts provide context for the baseline risk and offer insight into the stability and limitations of the predictive models presented in the study.

### 3.2. Implicit Measures as Predictors of SITB

Regarding implicit measures, the mean D/S-IAT and SI-IAT scores were −0.129 and 0.266, respectively. Correlation analysis revealed a significant positive correlation between the SI-IAT and total Columbia- C-SSRS (r = 0.279, *p* = 0.039), whereas the D/S-IAT showed no significant correlation (r = 0.002, *p* = 0.349) (see Table 2).

Predictive analyses of SITB outcomes at six months showed that neither the D/S-IAT nor the SI-IAT significantly predicted self-injurious behavior, thoughts of suicide, or suicide attempts. Odds ratios for these implicit measures ranged from 0.38 to 2.28 but did not reach statistical significance (all *p* > 0.38) (see Table 3).

### 3.3. Explicit Measures and Sociodemographic Predictors of SITB

In contrast, explicit measures showed significant associations with SITB outcomes. Odds ratios (OR) for continuous predictors are reported per 10-point increase to facilitate interpretation. Elevated scores in DERS scores were significantly related to increased risk of suicidal thoughts (OR = 1.45, 95% CI [1.14–1.82], *p* = 0.003) and self-injurious (OR = 1.30, 95% CI [1.03–1.63], *p* = 0.027). Similarly, increased impulsivity measured by BIS-11 predicted a greater risk of both suicide thoughts (OR = 1.60, 95% CI [1.07–2.37], *p* = 0.020) and self-injurious (OR = 1.49, 95% CI [1.01–2.16], *p* = 0.044). Specific facets of impulsivity, including cognitive and non-planning impulsivity, were also significantly associated with suicidal thoughts. Childhood trauma, assessed using the CTQ-SF, was significantly associated with suicidal thoughts (OR = 1.91, 95% CI [1.17–3.11], *p* = 0.008), but not with other SITB outcomes (see Table 4).

Among the sociodemographic variables analyzed, parental divorce was significantly associated with suicidal thoughts (OR = 7.741, 95% CI [1.72–34.79], *p* = 0.008) and showed a trend toward association with suicide attempts (OR = 5.143, *p* = 0.059). Previous suicide attempts significantly doubled the risk of recurrence of self-injurious (OR = 1.903, *p* = 0.049) (see Table 4).

Furthermore, correlation analyses revealed significant positive relationships between C-SSRS scores and BIS-11 (r = 0.537, *p* < 0.001) and DERS (r = 0.507, *p* < 0.001). This indicates that these explicit measures are closely related to the severity of SITB.

Overall, while implicit measures did not significantly predict SITB recurrence at six months, explicit measures of impulsivity, emotion dysregulation, childhood trauma, and certain sociodemographic factors emerged as significant predictors of suicidal ideation and self-injurious behavior in this adolescent sample.

## 4. Discussion

### 4.1. Summary of Results

This study examined the predictive value of the D/S-IAT and the SI-IAT in predicting the occurrence of SITB within six months, in a sample of adolescents with a recent history of SITB. Associations between SITB and risk factors such as emotion dysregulation, impulsivity, childhood trauma and sociodemographic variables were also explored.

Neither the SI-IAT nor the D/S-IAT were statistically significantly associated with an increased risk of SITB six months later. However, robust and statistically significant associations were found between measures of emotion dysregulation and impulsivity with SITB. There was also a trend toward significance between childhood trauma and suicidal thoughts. Parental divorce was related to future suicidal thoughts, while previous suicide attempts were associated with subsequent self-injurious. Our findings highlight that, within this high-risk adolescent group, explicit measures of emotion dysregulation and impulsivity are more reliable predictors of SITB recurrence than implicit cognitive measures over the six-month period. The limited predictive value of IATs for this high-risk, clinically acute sample of adolescents is likely due to the significant impact of explicit, proximal clinical states, such as emotional dysregulation and impulsivity, on short-term suicidal outcomes. Therefore, while IATs have theoretical potential, they may offer only modest incremental utility in acute contexts. By contrast, interventions that target emotion regulation and impulsivity mechanisms appear to offer a more immediate and practical approach to mitigating suicide risk among these vulnerable young people.

### 4.2. Comparison with Previous Research

Our findings contrast with those of Moreno et al. (2020) [14], who found a positive correlation between D/S-IAT scores and suicidal thoughts in adults. This discrepancy may be due to several methodological differences. Firstly, our sample consisted exclusively of adolescents, which suggests that the applicability of IATs may differ across developmental stages. Secondly, our sample size was smaller (*n* = 38 vs. *n* = 75) and our participants were inpatients, unlike the outpatients in Moreno et al.’s sample. It is plausible that greater clinical severity influences the implicit cognitive processes measured by the IAT, thereby affecting its predictive validity.

In contrast to studies by Nock et al. (2007) and Glenn et al. (2019) [15,16], our study found no significant predictive capacity for IATs. This could be attributed to the high baseline risk inherent in our sample, composed exclusively of adolescents with recent SITB, which might reduce the discriminatory power of implicit measures in such clinically severe populations. Moreover, some recent literature suggests that the predictive utility of implicit measures may be context-dependent and may not surpass explicit self-report instruments when those are comprehensive and precise [25,26,27].

Interestingly, the SI-IAT showed a significant correlation with the total C-SSRS score. This may indicate that adolescents engage in self-injurious behaviors as an ineffective strategy for managing psychological distress rather than as an immediate manifestation of death-related thoughts [18]. This could explain why the implicit association with death was more diffuse in our adolescent sample.

Regarding emotion dysregulation, our findings are consistent with previous research indicating that difficulties in regulating emotions mediate the relationship between emotional distress and suicide risk [9,11,12]. Emotion dysregulation may amplify the intensity of psychological pain, providing an indirect pathway between emotional suffering and suicidal behavior [11,12]. This conceptualization clarifies that the DERS captures deficits in emotion regulation rather than psychological pain itself, thereby reinforcing the construct validity of the variable assessed in this study [9].

As for psychological pain, the observed association between psychological pain and suicide risk aligns well with its mediating role as extensively reported in the literature [11,12]. Prior studies have demonstrated that elevated psychological pain corresponds with increased emotional suffering and a higher likelihood of SITB, particularly among adolescents [28,29]. Our results suggest that the DERS may serve as an indirect marker of severe psychological pain and vulnerability to suicide.

Further analysis of the DERS subscales revealed that emotional rejection, emotional interference, and emotional confusion were the dimensions most closely associated with suicidal thoughts. These maladaptive emotional processes are consistent with earlier findings that identify them as core components of psychological pain [11,12]. Difficulties in accepting negative emotions (emotional rejection), perceiving emotions as disruptive to daily functioning (interference), and lacking emotional clarity (confusion) likely amplify subjective suffering, thereby increasing suicide risk.

These emotional dysregulations may interact with other vulnerability factors, such as impulsivity, to intensify psychological pain and encourage self-harm or suicidal thoughts. Our findings related to psychological pain and impulsivity are consistent with prior research [9,17], confirming these factors as mediators between childhood trauma and SITB. Notably, impulsivity—particularly non-planned impulsivity—was associated with self-injurious and suicidal thoughts but not suicide attempts, replicating meta-analytic evidence linking this trait primarily to less lethal suicidal behaviors.

Our findings suggest a significant trend between childhood trauma and suicidal thoughts, which strengthens the evidence that adverse childhood experiences are a critical risk factor for SITB adolescents. Childhood trauma has been consistently linked to the dysregulation of emotional and cognitive processes, which may increase vulnerability to SITB via pathways such as increased psychological pain and impulsivity [17,30]. Adverse experiences such as abuse, neglect and exposure to familial suicidal behavior can lead to the development of maladaptive cognitive and emotional patterns, including feelings of hopelessness and self-blame, which can increase the risk of suicide [31,32]. Exposure to trauma may initiate a cascade of interactions between neurobiological, psychological and social factors that impair emotional regulation and heighten impulsivity [17]. Our results support this, as they link these traits to SITB recurrence. Therefore, childhood trauma should be considered a fundamental and pervasive factor in the development of suicidal behavior in adolescents. Integrating trauma-informed approaches into assessments and interventions is essential for mitigating risk and improving outcomes for vulnerable young people.

From a clinical perspective, these findings have direct implications for the assessment and prevention of suicide risk in adolescents. The prevalence of explicit, proximal indicators, such as emotion dysregulation and impulsivity, suggests that these constructs should be prioritized in clinical evaluations and therapeutic interventions, particularly in acute inpatient settings. While the limited contribution of IATs in this context is notable, this does not diminish their potential value. Rather, it highlights the need to refine and validate these implicit paradigms in less severe or community-based samples, where automatic self-associations may play a more prominent role.

When comparing our results with prior research, differences in methodological validity (e.g., outpatient versus inpatient recruitment, sample size, gender distribution and analytical strategy) must be considered as potential explanations for the heterogeneity of findings across studies. As 94.7% of our sample were female, these results also emphasize the importance of adopting gender-sensitive approaches to suicide prevention and promoting programs that strengthen emotion regulation and impulse control among adolescent girls, who exhibit higher rates of self-injurious behaviors.

Beyond clinical practice, these results emphasize the importance of integrating emotion regulation and impulsivity training into educational and public health programs, as early intervention in these areas could prevent suicidal vulnerability from escalating. Collaboration between the clinical, educational and policy sectors is essential to translate these findings into effective prevention strategies.

### 4.3. Strengths and Limitations

This study has several strengths. The prospective design, which included a six-month follow-up period, enabled the exploration of temporal relationships between risk factors and the onset of SITB. Including adolescents at high clinical risk, and conducting a multimodal assessment using implicit and explicit measures, as well as sociodemographic variables, provides a comprehensive view of the factors involved.

However, certain limitations must be considered. The small sample size (*n* = 38) and the fact that 94.7% of participants were female limit the statistical power and generalizability of the results to male and community-based adolescent populations. The hospitalized sample may skew the results towards more severe clinical profiles, limiting their applicability to outpatient settings. These characteristics could also partly explain the absence of significant predictive effects for IATs, which might show stronger associations in more heterogeneous or less clinically acute samples. Furthermore, collecting follow-up information exclusively from medical records may underestimate the actual incidence of SITB, as unrecorded episodes are excluded. Although no data on contact or visit coverage outside hospital records were available, this limitation introduces the potential for under-ascertainment and differential follow-up. To improve completeness, future studies should combine record review with structured follow-up interviews. Finally, the absence of a direct, validated measure of psychological pain restricts our ability to interpret its role as a mediator. However, its indirect presence can be inferred from the findings on emotional suffering. Furthermore, as multiple analyses were conducted without formal multiplicity corrections, the results should be treated as exploratory and interpreted with caution to avoid inflated type I error rates.

### 4.4. Future Lines of Research

Future research should increase the sample size and include a greater proportion of males, as well as diversifying the recruitment context. Incorporating an outpatient population would enable us to assess whether implicit measures demonstrate greater predictive power in less severe settings. Using specific instruments to measure psychological pain would enable us to analyze its mediating role in SITB. It would also be interesting to explore models that combine implicit and explicit measures, in order to determine whether integration improves the accuracy of risk prediction. Furthermore, it would be valuable to develop and validate combined predictive models integrating implicit and explicit measures alongside sociodemographic and clinical variables. This could be achieved by applying advanced analytical techniques, such as machine learning and multilevel longitudinal study designs. Such models could improve the accuracy and generalisability of suicide risk prediction by capturing complex interactions and temporal dynamics across different adolescent populations and settings.

## 5. Conclusions

While implicit measures were not statistically significant in predicting SITB recurrence at six months, meaningful associations were identified between outcomes such as suicidal thoughts and self-injury, and explicit measures, particularly those assessing emotion dysregulation and impulsivity. These findings emphasize the greater predictive value of clinical measures in high-risk adolescent populations, while suggesting that implicit tests may require methodological refinement or larger sample sizes to demonstrate their potential usefulness. Furthermore, these results highlight the urgent need to integrate emotion regulation-based interventions into clinical practice to reduce suicide risk among adolescents. By providing a prospective analysis of implicit measures, this study makes a valuable contribution to the growing body of research on suicide risk. It advances our understanding of the mechanisms involved in SITB and highlights the importance of preventive interventions for vulnerable young people. Lastly, further validation studies of the IATs within Spanish adolescent populations are essential to ensure their psychometric robustness and cultural relevance for effective clinical application, reinforcing the importance of targeted preventive interventions for vulnerable young people.

## Figures and Tables

**Table 1:** Sociodemographic and clinical characteristics of the sample (*n* = 38).

Variable	*n* (%)	Mean ± SD	Range
Female	36 (94.7)		
Age (years)		14.92 ± 1.46	12–17
LGTBIQ+	3 (7.9)		
Divorce	21 (55.2)		
Family conflict	22 (57.9)		
First-generation migrant	7 (18.4)		
Second-generation migrant	20 (52.6)		
Low socioeconomic status	19 (50.0)		
History of abuse	16 (42.1)		
History of SITB	33 (86.8)		
Family history of mental illness	31 (81.6)		
Eating disorders	16 (42.1)		
Substance use	6 (15.8)		
Previous psychiatric admissions	13 (34.2)		
Number of previous admissions		0.63 ± 1.21	0–5
Main psychiatric diagnosis			
Mood disorders	20 (52.6)		
Eating disorders	3 (7.9)		
Other	15 (39.5)		
Type of suicidal attempt			
Overdose	9 (23.7)		
Cutting	2 (5.3)		
Poisoning	1 (2.6)		
Previous follow-up			
None	8 (21.0)		
Psychiatry	1 (2.6)		
Psychology	1 (2.6)		
Combined	28 (73.7)		

**Table 2:** Correlation between IATs and C-SSRS.

	R de Pearson	95% CI	*p*
SI-IAT	0.279	0.020–1.000	0.039
D/S-IAT	0.002	−0.259–1.000	0.349

CI = Confidence Interval; D/S-IAT = Death/Suicide Implicit Association Test; SI-IAT = Self-Injury Implicit Association Test.

**Table 3:** IAT as a predictor of SITB at 6 months.

	Self-Injurious at 6 Months			Suicidal Ideation at 6 Months			Suicide Attempts at 6 Months		
	OR	95% CI	*p*	OR	95% CI	*p*	OR	95% CI	*p*
SI-IAT	0.506	0.092–2.78	0.433	1.96	0.33–11.59	0.458	1.246	0.175–8.873	0.826
D/S-IAT	2.277	0.35–14.48	0.383	0.544	0.077–3.84	0.542	0.380	0.043–3.391	0.387

CI = Confidence Interval; D/S-IAT = Death/Suicide Implicit Association Test; SI-IAT = Self-Injury Implicit Association Test.

**Table 4:** Risk factors associated with SITB at 6-month follow-up.

	Self-Injurious at 6 Months			Suicidal Ideation at 6 Months			Suicide Attempts at 6 Months		
	OR	95% CI	*p*	OR	95% CI	*p*	OR	95% CI	*p*
Divorce	1.500	0.44–5.10	0.516	7.741	1.72–34.79	0.008	5.143	0.940–28.138	0.059
Previous suicide attempts	1.903	1.003–3.61	0.049	-	-	-	-	-	-
DERS	1.30	1.03–1.63	0.027	1.45	1.14–1.82	0.003	1.17	0.91–1.52	0.207
BIS-11 Cognitive Motor Non-planning	1.49 1.14 1.06 1.62	1.01–2.16 1.02–1.27 0.99–1.14 1.11–2.28	0.044 0.126 0.105 0.032	1.60 1.18 1.15 3.08	1.07–2.37 1.00–1.35 1.02–1.22 1.27–7.30	0.020 0.046 0.999 0.013	1.24 - - -	0.87–1.75 - - -	0.300 - - -
CTQ-SF	1.91	1.17–3.11	0.109	1.91	1.17–3.11	0.008	1.11	0.84–1.44	0.485

BIS-11 = Barratt Impulsiveness Scale; C-SSRS = Columbia-Suicide Severity Rating Scale; CTQ-SF = Childhood Trauma Questionnaire–Short Form; DERS = Difficulties in Emotion Regulation Scale; OR = Odds Ratio.

## Data Availability

The data that support the findings of this study are available from the author upon reasonable request.

## Abbreviations

The following abbreviations are used in this manuscript:

SITB	Self-Injurious Thoughts and Behaviors
IATs	Implicit Association Tests
D/S-IAT	Death/Suicide IAT
SI-IAT	Self-Injury IAT
C-SSRS	Columbia Suicide Severity Rating Scale
DERS	Difficulties in Emotion Regulation Scale
BIS-11	Barratt Impulsiveness Scale
CTQ-SF	Childhood Trauma Questionnaire —Short Form
NSSI	Non-Suicidal Self-Injury
EU	European Union
